# Explorations of Coast Survey Bureau

**Published:** 1871-12

**Authors:** 


EXPLORATIONS BY THE COAST SURVEY BUREAU.—Prof. Agassiz, says the *American Naturalist*, has accepted an invitation to take passage in the iron Coast Survey steamer, which has just been built near Wilmington, Del., and which sails for the Pacific coast in September next. The expedition will take deep-sea soundings all the way. Secretary Boutwell has written to the Secretaries of State and Navy, asking that naval and other officers may be instructed to afford such courtesy and assistance to the exploring party as may be desirable.

Count Courtales, Rev. Dr. Hill, and Dr. W. White, of Philadelphia, will accompany the expedition.—*N. Y. Med. Record.*

